# Migrating transient osteoporosis of the hip in a 30-year-old man

**DOI:** 10.4103/0019-5413.50872

**Published:** 2009

**Authors:** SS Suresh, John K Thomas, Sameer Raniga

**Affiliations:** Department of Orthopaedics, Ibri Regional Referral Hospital, PO Box 46, Ibri 516, Sultanate of Oman; 1 Department of Orthopaedics, Sultan Qaboos University Hospital, PO Box 35, Al-Khod123, Muscat, Sultanate of Oman; 2 Department of Radiology, Al-Afaq MRI Centre, PO Box 794, PC 117, Al Wadi Al Kabir, Sultanate of Oman

**Keywords:** Alendronate, hip, magnetic resonance imaging, osteoporosis, pregnancy

## Abstract

Transient osteoporosis of hip is a condition of unknown etiology, presenting as painful limping, and characterized by osteopenia of the involved joint without preexisting disease or immobilization. Most of the cases were reported in middle-aged men, and one-third of the cases develop in women in the third trimester of pregnancy. The hypothesis that this condition leads to avascular necrosis of the hip has been disproved by various reports and hence does not warrant any surgical interference. This is a self limiting condition, which needs regular follow-up. The authors report a case of migrating transient osteoporosis of the hip in a 30-year-old man successfully treated with antiresorptive treatment.

## INTRODUCTION

Transient osteoporosis of hip (TOH) or bone marrow edema syndrome (BMES) is a condition of unknown etiology, though many unproved theories have been proposed, affecting women in the third trimester of pregnancy and middle-aged men. The condition is often self-resolving with conservative management, though there are reports of fracture neck of femur and recurrent episodes.^1^–^4^ The term regional migratory osteoporosis (RMO) has been used when there is transient osteoporosis, with typical migratory features with involvement of another joint, which typically occurs within 6 months of onset of primary symptoms.^2^,^5^–^8^ RMO typically migrates to adjacent joints and most commonly affects the knee and ankle. Conservatism is the mainstay of treatment but the duration of symptoms can be reduced by oral or intravenous bisphosphanates as per various reports.^1^,^2^,^9^–^11^

## CASE REPORT

A 30-year-old man was seen in the Orthopaedic clinic with pain left hip after prolonged traveling. He was of moderate built and had no significant medical history and was not on any medications. He was not addicted to alcohol and never smoked. There was terminal painful restriction of flexion and abduction.

**Figure 1:**
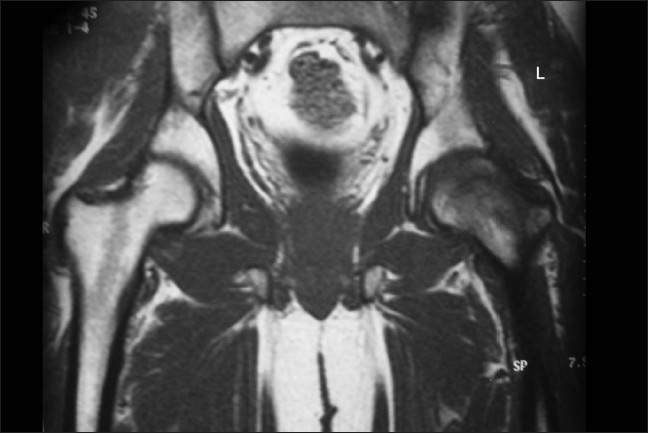
Coronal T1-weighted image shows homogeneously hypointense marrow edema with medial sparing of the left femoral head

**Figure 2:**
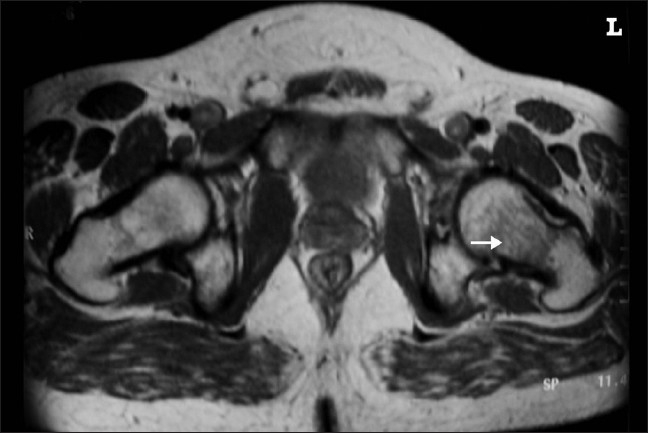
PD transverse T2-weighted image of left hip showing bone marrow edema (arrow)

**Figure 3:**
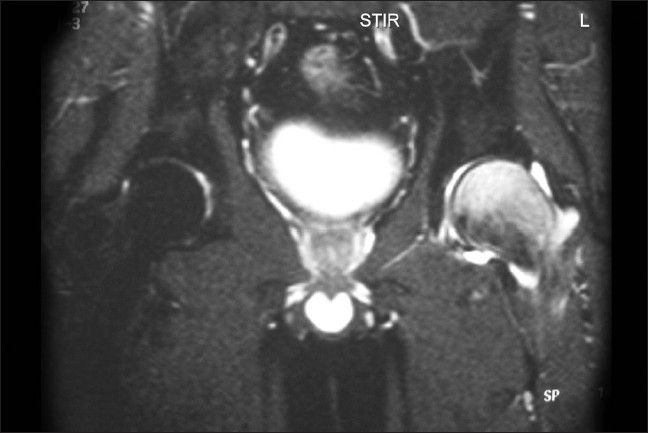
Coronal STIR images shows marrow edema as uniform hyperintensity signal involving head and neck of left femur and extending till the intertrochanteric region. No evidence of avascular necrosis of left femoral head

He had no neurological deficit, and there were no signs related to the lumbar disc prolapse. X-rays of the pelvis done on initial presentation were normal. Blood parameters including C-reactive protein, ESR, and liver function tests were normal. Clinically, he was found to have normal hormonal function, and his thyroid function tests were also normal. Ultrasound scanning of the abdomen was normal and didn't show hepatomegaly. Ultrasound scanning of the left hip revealed normal study. He was advised non-weightbearing on the left hip. Further review in 2 weeks the X-rays showed osteopenia of the head and neck of left femur, and the diagnosis of transient osteoporosis of the hip (TOH) was thought of and MRI was done. MRI showed marrow signal abnormality (diffusely hypointense signals on T1W images and hyperintense signals on T2 and FAT saturation images), which was extending in the femoral head and neck till the intertrochanteric region [Figures 1–3].

There was minimal effusion in the hip as well. He was subjected to a Tc99 methylene diphosphanate bone scan, which showed increased uptake in the left hip and knee joints. He was managed by non-weightbearing on the left leg and analgesics (diclofenac sodium slow release 100 mg tablets). The patient became asymptomatic after 4 months of treatment and remained symptom-free for 14 months.

**Figure 4:**
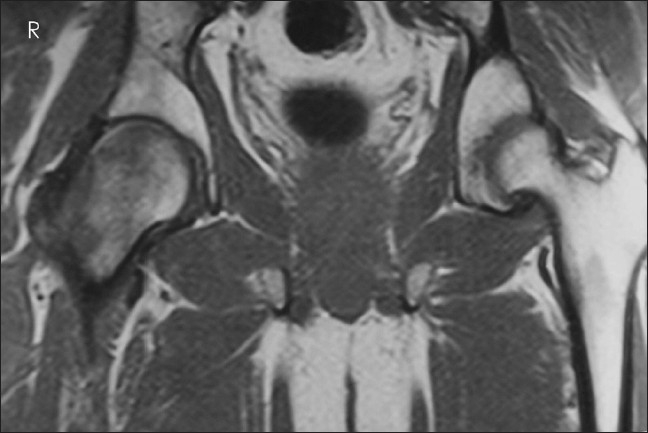
Coronal T1-weighted image shows homogeneously hypointense marrow edema, with normal left hip

**Figure 5:**
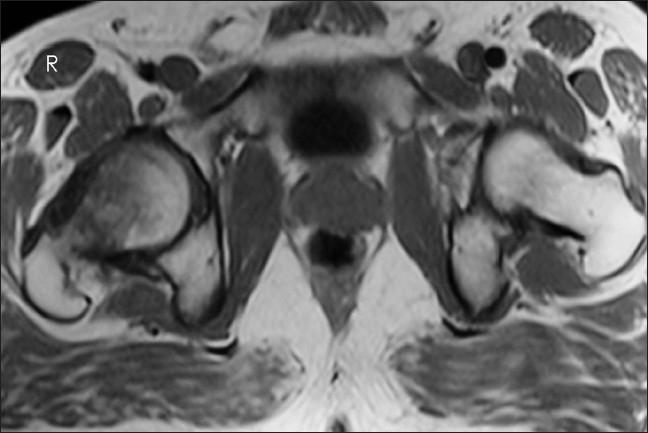
T1-weighted transverse image showing the bone marrow edema head and neck of right femur

**Figure 6:**
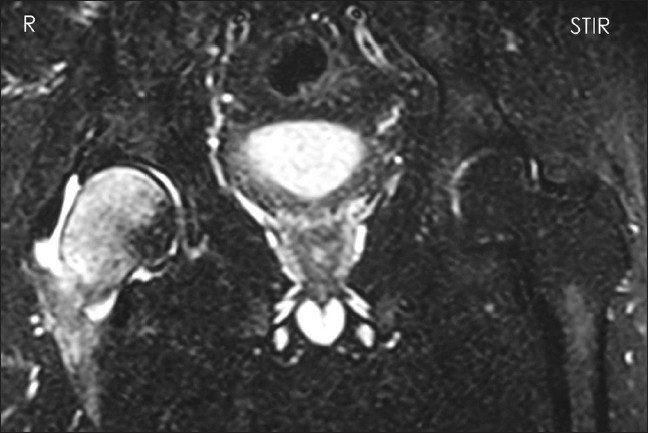
Coronal STIR (T2W) image showing same appearance as in Figure 3 and normal left hip

After 14 months of presentation, he developed pain in his right hip, with terminal restriction of range of movements. X-rays of the hip done this time showed osteopenia of the right proximal femur. Repeated MRI scan showed the same findings as in the previous MRI, with abnormal signal intensities but sparing some parts of the head of femur inferomedially. The previously affected left hip was found normal in this MRI [Figures 4–6].

The patient was started on calcitonin nasal spray and alendronate (Fosamax 70 mg tab once weekly). Calcitonin was initiated for 6 weeks and the oral weekly bisphosphanates were given for 3 months. The symptoms resolved this time within one month of starting the antiresorptive treatment. Follow-up at one year showed complete resolution of the osteopenia and the patient was symptom-free.

## DISCUSSION

First described by Curtiss and Kincaid in 1959 as transitory demineralization of the hip in pregnancy, TOH is a self limiting condition, which resolves without leaving any residual effects.^12^ Subsequent to this there are many reports in the English literature,^3^,^4^,^7^ mostly as small series. But most of the cases remain unreported due to the lack of awareness or reluctance on the part of pregnant patients to undergo imaging.

The presentation in the hip is dull aching pain in the hip progressing over a period of time, with terminal limitation of movements. Hip is classically involved, but there are reports of involvement of the knee, ankle, and the foot.^10^

The clinical and radiological features are evaluated in three phases. Phase one, which lasts for 1–2 months, when there is increasing hip pain. The next phase, which shows osteopenia in the proximal femur, lasts for 2–3 months and clinical symptoms becomes more predominant. The third phase, which extends to 6 months, after emergence of the symptoms starts to regress.^13^,^14^ Patients with subchondral fracture take longer time to recover. Bilateral incidence of 20% is reported in a study from Japan.^15^

Many theories are proposed, including endocrine dysfunction, local compression of the vessels, and nerves by the gravid uterus,^16^ and mechanical compression of the obturator nerve and reduction in arterial perfusion to the joints.^12^ But the proposed theories do not explain the occurrence of TOH in men. BMES may be due to temporary ischemic hypoxia of the femoral head resulting in vascular dilatation, increased perfusion, and interstitial edema.^17^ Authors conclude that the increased perfusion may result in transient demineralization of the proximal femur. The etiology of TOH is still not clear though many theories have been proposed.

Initial X-rays are most of the times normal because it takes time for the osteopenia to develop. Characteristic radiographic findings are present within 4–8 weeks after onset of symptoms. There is often a delay in diagnosis as the X-rays are normal in the initial period but if suspected an MRI can confirm the diagnosis. Once the disease progresses, the proximal femur including the head and neck up to the intertrochanteric area gets demineralized with preserved joint space.^16^

MRI is diagnostic and excludes other causes of hip pain in the adult. MRI shows characteristic features of bone marrow edema as early as 48 hours after the onset of pain.^5^,^18^,^19^

T1-weighted images show ill-defined area of decreased signal intensity when compared with normal intensity of the normal bone marrow in the proximal femur. In T2-weighted images increased signal intensity was observed.^18^ The T2-weighted images may also show the joint effusion. The signal intensity of marrow is not altered in other forms of osteoporosis.^18^

The MRI appearance of TOH was first described by Bloem.^18^ Transient BME syndrome is the term used for patients in whom reversible BME pattern is seen in MR images without radiological evidence of osteopenia.^20^

Radiological appearance of TOH and AVN may be strikingly similar,^5^ resulting in unnecessary surgical interventions for this self limiting condition.

It is possible to differentiate TOH and AVN of head of femur from the plain X-rays and MRI. In TOH, there is diffuse osteopenia of the entire femoral head and neck, without articular erosion or subchondral collapse, unlike in AVN, where the appearance is of mottled radiolucent area surrounded by an area of sclerosis. There is segmental involvement in the anterosuperior subchondral area of the head of femur in AVN.^19^ MRI shows mottled low signal lesion in T1W images in AVN and a high signal lesion in the bone marrow suggesting marrow edema on T2 W images.^15^,^18^,^19^ There are reports disproving the relation of TOH and avascular necrosis.^5^,^17^

The mainstay of treatment is conservative with protective weightbearing, to prevent pathological fractures.^1^,^2^,^14^,^15^ Protective weightbearing is advised during the period of osteopenia to prevent pathological fracture of the neck of femur.^4^,^21^

Oral, intravenous bisphosphanates and calcitonin have been used by various authors with beneficial effects.^1^,^2^,^9^,^10^,^16^ The authors considered calcitonin intranasal spray and weekly oral bisphosphanate since the condition developed in the other hip after complete resolution of the TOH in left hip. Improvement was noted within 2 weeks of treatment with oral bisphosphanate in various reports.^10^,^11^

Varenna *et al.*^9^ used 45 mg of pamidronate diluted in 500 mL of isotonic saline intravenously, once every 3 days as three infusions. Clinical recovery was observed in one month and MRI became normal in 3 months.^9^ The possible effect of pamidronate is as an anti-inflammatory agent.

Core decompression has been performed to prevent progression to osteonecrosis since many authors thought TOH as a precursor of avascular necrosis of head of femur,^1^ but this is unwarranted procedure as there are various studies, which proved the benign nature of the TOH.^5^,^17^,^19^ Patients can be managed conservatively without surgical intervention and needs to be followed up periodically with imaging.^1^ Conservative treatment in the form of protected weightbearing, intermittent traction, and analgesics, remains the mainstay of management, but oral bisphosphanates can minimize the duration of symptoms.

Complete resolution takes 2–24 months as per various reports.^1^,^9^,^11^,^18^ Diwanji recommends follow-up for a period of 24 months.^1^ Ergun^15^ found direct correlation between the extent of proximal femoral edema and the duration of recovery period. The recovery as per authors gets prolonged if there is a larger subchondral fracture area as seen in the MRI. Migratory recurrence of symptoms occurs within 2 years after pain relief.^21^

TOH may have migratory features and both regional migratory osteoporosis (RMO) and TOH are considered part of the same spectrum.^2^,^6^,^7^ RMO predominantly involves the ankle, foot, and knee, and less common for hip involvement,^2^ and it usually recurs in adjacent joints. RMO is diagnosed when there is transient osteoporosis, with typical migratory features with involvement of another joint, which typically occurs within 6 months of onset of primary symptoms.^6^ Secondary joint involvement occurs soon after the pain from the primary joint reaches its peak or resolves.^6^ In our case, the patient was symptom free for 14 months, and hence, we consider the condition as repeated episode of unifocal disease.^8^ In the series of Balakrishnan *et al*.,^5^ two patients were found to have recurrent TOH, with the second joint affected 5 and 3 months after resolution of symptoms in the primary joint. The clinician should be aware of the possibility of recurrence of this condition in another joint. Shifrin reported three patients who had recurrent TOH after 1, 2, and 4 years.^3^ TOH is often under diagnosed, as most of the cases which occur during pregnancy are not proved as X-rays are not done for fear of damage to fetus.

TOH should be considered in the differential diagnosis of painful limping in men and properly treated to prevent development of the disease in the contralateral hip. The authors report a case of migrating TOH after complete resolution in the previously affected side, successfully treated with antiresorptive measures.
